# m^6^A RNA Methylation Regulators Contribute to Eutopic Endometrium and Myometrium Dysfunction in Adenomyosis

**DOI:** 10.3389/fgene.2020.00716

**Published:** 2020-07-03

**Authors:** Junyu Zhai, Shang Li, Sushmita Sen, Jessica Opoku-Anane, Yanzhi Du, Zi-Jiang Chen, Linda C. Giudice

**Affiliations:** ^1^Center for Reproductive Sciences, Department of Obstetrics, Gynecology and Reproductive Sciences, University of California, San Francisco, San Francisco, CA, United States; ^2^Center for Reproductive Medicine, Ren Ji Hospital, School of Medicine, Shanghai Jiao Tong University, Shanghai, China; ^3^Shanghai Key Laboratory for Assisted Reproduction and Reproductive Genetics, Shanghai, China

**Keywords:** adenomyosis, m^6^A, METTL3, endometrium, myometrium, *in silico*

## Abstract

Adenomyosis is a prevalent, estrogen-dependent uterine disorder wherein endometrial cells are abnormally present in the myometrium and are surrounded by hyperplastic/hypertrophic smooth muscle. Its etiology is unclear, although endometrial cell invasion into the myometrium has been postulated. RNA methylation, particularly N6-methyladenosine (m^6^A), plays an important role in regulating various physiological processes and invasive disorders. The goal of this *in silico* and lab-based experimental study was to explore a possible role for m^6^A in adenomyosis. Gene expression profiles of both the endometrium and myometrium of women with adenomyosis (cases) and without disease (controls) were obtained from the publicly available Gene Expression Omnibus (GEO) database. In the endometrium, STRING database analysis revealed that *METTL3* functions as a “hub” gene of m^6^A RNA methylation regulators, and the genes involved in m^6^A regulation, including *METTL3*, *FTO*, *ZC3H13*, and *YTHDC1* expression, were significantly decreased in cases versus controls. Functional, co-expression, and correlational analyses of endometrium from cases versus controls revealed decreased total m^6^A levels, induced by *METTL3*, and the downstream elevated *insulin−like growth factor−1(IGF1)* and *D-Dopachrome Tautomerase* (*DDT)*, with the latter two having known functions in epithelial proliferation and cell migration, which are important processes in the pathogenesis of adenomyosis in endometrium. m^6^A RNA methylation regulators, including *RBM15/15B*, *ALKBH5*, *FTO*, *YTHDF1/2*, *KIAA1429*, *HNRNPC*, *METTL3*, *ZC3H13*, and *YTHDC2*, were also differentially expressed in the myometrium from cases versus controls. We validated decreased total m^6^A levels and differential expression of m^6^A RNA methylation regulators in the myometrium of patients with adenomyosis using qRT-PCR, immunohistochemistry and tissues available from our biorepository. Possible target genes, including *cadherin 3(CDH3)*, *sodium channel*β*-subunit 4* (*SCN4B)*, and *placenta-specific protein 8 (PLAC8)*, which are involved in cell adhesion, muscle contraction and immune response in the myometrium of adenomyosis patients were also validated. Thus, through extensive public database mining and validation of select genes, this study, for the first time, implicates m^6^A and its methylation regulators in the pathogenesis of adenomyosis. Follow on functional studies are anticipated to elucidate mechanisms involving m^6^A and its regulators and down-stream effectors in the pathogenesis of this enigmatic reproductive disorder and potentially identify druggable targets to control its associated symptoms.

## Introduction

Adenomyosis is a common disease of the uterus in which endometrial epithelial cells and stromal fibroblasts abnormally are found in the myometrium, wherein they elicit hyperplasia and hypertrophy of surrounding smooth muscle cells (Bird et al., 1972). It occurs in 8–27% of reproductive age women (Kissler et al., 2008) and results in a diffusely enlarged uterus, pelvic pain, heavy menstrual bleeding and infertility in those affected (Benagiano et al., 2012). Historically, definitive diagnosis was based on histological examination of hysterectomy specimens. Currently, decreased echogenicity or signal intensity on ultrasound and magnetic resonance imaging (MRI), respectively, is commonly used to diagnose adenomyosis which can occur in a diffuse pattern, as discrete adenomyomas, or cystic lesions in the uterine smooth muscle layer (Reinhold et al., 1998). As mechanisms underlying the pathogenesis and pathophysiology of adenomyosis are not well understood, therapies are inadequate to control symptoms or to facilitate successful pregnancy (Li et al., 2018).

Disruption of the “inner myometrium,” i.e., the normal boundary between the endometrial basal layer and the myometrium, has been postulated to underlie adenomyosis pathogenesis, with subsequent endometrial tissue and cells migrating into the adjacent smooth muscle compartment (Vercellini et al., 2006). As adenomyosis is more prevalent in women with previous cesarean section, it has been postulated that the endometrium invades a predisposed myometrium or a traumatized endometrial-myometrial interface during periods of regeneration, healing, and reepithelization (Curtis et al., 2002). In addition, there also evidence of familial predisposition in which genetic, immunological and other factors are involved (Arnold et al., 1995). Abnormal Müllerian and mesenchymal interactions during uterine development also may contribute to its pathogenesis, and tissue injury typically activates adult stem cells, which may establish endometrial lineage cells through disruption of endometrial stem/progenitor cell niches (Gargett et al., 2016). Thus, both compartments (endometrium and myometrium) have been implicated in the pathogenesis of adenomyosis, although more research is required to understand, mechanistically, the initiation and progression of the disease. Herein, we focus on RNA methylation in both compartments.

Epigenetic modifications play an important role in regulation of human physiology and invasive diseases, among which DNA and RNA methylation are involved. While most studies have focused on the role of DNA methylation in the female reproductive system, less data are available regarding RNA methylation, especially in adenomyosis. N6-methyladenosine (m^6^A) is the most abundant modification on mRNAs (Zaccara et al., 2019). m^6^A provides dynamic regulation of the nucleation, splicing, translation and stability of mRNA molecules (Wang et al., 2014; Roundtree and He, 2016; Deng et al., 2018), thereby influencing fundamental biological and pathological processes such as proliferation, differentiation, cellular response to stress and tumorigenesis (Peer et al., 2017). The m^6^A modification regulators are classified into the three groups: writers, erasers and readers (Wu et al., 2020). “Writers” include the m^6^A methyltransferases that promote methylation of m^6^A. They are mainly composed of methyltransferase−like 3 (METTL3) and 14 (METTL14) and Wilms’ tumor 1−associating protein (WTAP). Moreover, KIAA1429, zinc finger CCCH−type containing 13 (ZC3H13), METTL16 and RNA binding motif protein 15/15B (RBM15/15B) are also contribute to the RNA methylation (Wen et al., 2018; Yue et al., 2018). “Erasers” are demethylases which consist of fat mass and obesity−associated protein (FTO) and AlkB homolog 5 (ALKBH5). “Readers” are proteins that bind to the m^6^A site and promoting the function of m^6^A. They are predominantly in the YT521−B homology (YTH) protein family [YTH domain family 1/2/3 (YTHDF1/2/3) and YTH domain containing 1/2 (YTHDC1/2)], nuclear heterogeneous protein HNRNP family and IGF2BP protein family (Liao et al., 2018).

Previous studies indicated that m^6^A RNA methylation mediates cell proliferation and apoptosis in different cell types (Gu et al., 2018). Notably, increased proliferation and inhibition of cellular apoptosis are characteristics of the endometrium in women with adenomyosis (Li et al., 2019). m^6^A also contributes to the epithelial-to-mesenchymal transition (EMT) of cancer cells (Li et al., 2020) and EMT is considered to be a possible mechanism for the transfer of epithelial cell into the myometrium in adenomyosis patients (Hu et al., 2020). Moreover, m^6^A and its methylation regulators also regulate T cell activity (Winkler et al., 2019) and vascular development, which is involved in endometrial dysfunction of adenomyosis patients (Ota et al., 1998; Benagiano and Brosens, 2012). Thus, m^6^A RNA methylation may also play a role in the pathogenesis of adenomyosis.

m^6^A and its methylation regulators can also play roles in endometrial function in other settings. Liu et al. reported that about 70% of endometrial tumors exhibit reductions in m^6^A RNA methylation due to reduced *METTL3* expression. Moreover, m^6^A mRNA methylation is regarded as an oncogenic mechanism in endometrial cancer through regulation of AKT signaling (Liu et al., 2018). A previous study indicated that adenomyosis and type I endometrial cancer are linked to sex steroid action and exhibit gene expression profiling supporting a relationship between endometrial cancer and adenomyosis (Inoue et al., 2019), and women with adenomyosis are at higher risks of endometrial cancer (Yeh et al., 2018). The PI3K-AKT pathway, BCL2 apoptosis regulator and other factors are implicated in both adenomyosis and endometrial cancer (Roddy and Chapman, 2017). Thus, m^6^A RNA methylation may also contribute to endometrial dysfunction in women with adenomyosis.

Herein, we have investigated expression of m^6^A RNA methylation regulators in both endometrium and myometrium of women with versus without adenomyosis, providing a novel perspective and laying the foundation to elucidate underlying mechanisms of adenomyosis pathogenesis and pathophysiology.

## Materials and Methods

### Gene Expression Profile

We searched the associated gene expression profiles of the eutopic endometrium of adenomyosis patients in Gene Expression Omnibus (GEO) database^1^, using the keywords “adenomyosis”, “eutopic endometrium”, and “Homo sapiens.” We chose GSE78851 (Herndon et al., 2016) for analysis (5 control and 3 adenomyosis). All the eight samples of eutopic endometrium are in proliferative phase and we retained gene expression datasets from the Affymetrix Human Gene 1.0 ST Array (HuGene-1_0-st) and detected gene expression changes in the eutopic endometrium between three patients with adenomyosis and 5 healthy women (control).

We further searched the gene expression profiles of the myometrium of women with adenomyosis in GEO database using “adenomyosis,” “myometrium” and “Homo sapiens” and chose GSE7307 to investigate the mechanism of adenomyosis from the view of myometrium (10 women with adenomyosis versus 40 without adenomyosis). The gene expression was got from Affymetrix Human Genome U133 Plus 2.0 Array (HG-U133_Plus_2). We detected gene expression of myometrium between 10 women with adenomyosis (cases) and 40 without adenomyosis (controls).

### Identifying Differentially Expressed Genes (DEGs)

After downloading the GSE78851 and GSE7307 from GEO, the “impute” package of R software was used to impute the missing expression data while “limma” package was used to normalize the gene expression and identify the differentially expressed genes separately. The significant difference was defined as log FC > 1 and *P* < 0.05.

### Selection of m^6^A RNA Methylation Regulators

We first assembled a list of eighteen m^6^A RNA methylation regulators from published literature and review (Wu et al., 2020), and then we restricted the list to sixteen genes with available RNA expression data separately from the GSE78851 and GSE7307 in GEO dataset. This yielded a total of sixteen m^6^A RNA methylation regulators. Then, we systematically compared the expression of these sixteen m^6^A RNA methylation regulators in the eutopic endometrium and myometrium of women with and without adenomyosis separately using Wilcox test in R software (^∗^*p* < 0.05, ^∗∗^*p* < 0.01, ^∗∗∗^*p* < 0.001).

### The Correlation Between m^6^A RNA Methylation Regulators and DEGs

Interactions among m^6^A RNA methylation regulators were analyzed using the STRING^2^. Moreover, the correlation between m^6^A RNA methylation regulators and highly enriched Gene ontology (GO) terms related DEGs were identified using Spearman correlation in the “Corrplot” package of R software. *p* < 0.001 was considered as significantly correlated to each other.

### Weighted Gene Co-expression Network Analysis (WGCNA)

To verify the potential relationship and the co-expression genes of m^6^A RNA methylation regulators that were differentially expressed in the women with and without adenomyosis, we used another method to analyze the DEGs in the eutopic endometrium and myometrium separately. WGCNA assigns a connection weight to each gene pair in the network, being more meaningful compared to traditional methods that use binary information. Thus, WGCNA can be used to find modules of highly correlated genes and identify candidate target genes (Langfelder and Horvath, 2008). We used WGCNA to analyze the DEGs and identified the relationship between m^6^A RNA methylation regulators and the potential target genes of m^6^A RNA methylation regulators in the eutopic endometrium and myometrium separately.

### Enrichment Analysis

GO analysis was used to identify the possible molecular function and visualize the potential biological meaning behind DEGs, whereas Kyoto Encyclopedia of Genes and Genomes (KEGG) was to analyze the potential functions of these genes. The gene ID was set using the “org.Hs.eg.db” of R software, and then GO and KEGG pathway enrichment analyses were performed with “clusterProfiler.” *p* < 0.05 was identified as significant.

### Clinical Sample Collection

Clinical symptoms and histologic evaluation of hysterectomy specimens identified samples from cases and controls (latter had hysterectomies due to uterine fibroids or dysmenorrhea). Full thickness uterine specimens (including endometrium, inner myometrium and outer myometrium) were collected and stored in OCT at −80°C. All participants (*n* = 9 cases; *n* = 9 controls) were in the proliferative phase of the cycle, confirmed by endometrial histology (Noyes et al., 1950) and serum estrogen (E_2_), progesterone levels (P_4_). All participants were documented to be not pregnant and had not received hormonal or gonadotropin-releasing hormone agonist (GnRHa) therapies for at least 3 months prior to tissue sampling. 6 cases and 6 controls were used for the detection of the percentage of m^6^A in total RNA and validation of real-time polymerase chain reaction (qRT-PCR) while the rest of the cases and controls samples (3 for each group) were used for immunohistochemistry (IHC) and m^6^A quantification of only mRNA. The clinical samples were collected from the Endometrial Tissue and DNA Bank at the University of California, San Francisco under an approved human subjects protection protocol (IRB # 10-02786), after written informed consent of all participants.

### Hematoxylin and Eosin (H&E) Stain

The full thickness uterine tissue was embedded in OCT and stored at −80°C. Five μm-thick tissue frozen sections were prepared and then OCT blocks were returned to −80°C and stored at this temperature until further use. Slides were brought to room temperature and left for at least an hour prior to fixing the tissue in 100% ethanol for 10 min. Then, the slides were stained in hematoxylin for 7 min and differentiated by hydrochloric acid for 30 s. Finally, the sections were incubated in eosin for 1 min before covering the slide and visualizing using a microscope (Zeiss, Oberkochen, Germany).

### qRT-PCR

The endometrium and myometrium of OCT blocks were separated on ice according to H&E stain. We chose the myometrium close to the interface between the endometrium and myometrium, so that most of myometrial samples were mainly inner myometrium. Total RNA from endometrium and myometrium was extracted, separately, using an RNA isolation kit (Macherey-Nagel, Bethlehem, PA, United States) and reversely transcribed into cDNA (TAKARA, Dalian, China). The mRNA expression of target genes was detected using qRT-PCR. Results were analyzed by ΔΔCt method. The ratio of the target gene over β*-Actin* was calculated as the target mRNA level. The primer sequences used for targeting genes are shown in Supplementary Table.

### Total m^6^A RNA Methylation Assay

The m^6^A RNA Methylation Assay Kit (Abcam, Cambridge, United Kingdom) was used to evaluate the content of m^6^A in total RNA as protocol. Total RNA was bound to the strip wells using an RNA high binding solution provided by the manufacturer. Briefly, 200 ng total RNA were coated on each assay well, followed by specific capture with N6-methyladenosine antibody and the detection antibody. Then the detected signal was enhanced and quantified colorimetrically by reading the absorbance in a microplate spectrophotometer at a wavelength of 450 nm. The amount of m^6^A is proportional to the OD intensity measured. Finally, calculations using OD450 values were performed based on the standard curve to get the final content of m^6^A level in total RNA.

### IHC

Five μm-thick tissue frozen sections were prepared using OCT blocks of full thickness uterine tissue. Slides were rehydrated and then blocked using blocking buffer for 1 h at room temperature. Heat-mediated antigen retrieval was carried out with 10 mM sodium citrate, 0.05% Tween 20, pH 6, and then slides were incubated in anti-METTL3 antibody (1:200 dilution; Proteintech, Wuhan, China) overnight at 4°C. After being washed with PBS, the slides were processed with the secondary antibody (1:400) for 1 h at room temperature, and then the color reaction was visualized by exposure to diaminobenzidine (DAB). Slides were counterstained with hematoxylin and dehydrated through graded alcohols and xylene before visualizing using a microscope (Zeiss). Staining was assessed using Image J.

### m^6^A Quantification by Liquid Chromatography-Tandem Mass Spectrometry (LC-MS/MS)

RNA m^6^A quantification by LC-MS/MS was performed as described previously (Liu et al., 2014). In brief, total RNA from the endometrium and myometrium of women with and without adenomyosis was isolated using TRIzol reagent (Invitrogen, CA, United States), and polyadenylated RNAs were extracted by oligo d(T)25 magnetic beads (NEB, Ipswich, MA, United States), followed by removal of rRNA with RiboMinus Eukaryote Kit (Ambion, Austin, TX, United States). 200 ng mRNA were digested by nuclease P1 (1 U, Sigma-Aldrich, St. Louis, MO, United States) in 20 μL buffer which contained 25 mM NaCl, 2.5 mM ZnCl2 for 2 h at 37°C. After an additional incubation at 37°C for 2 h, the solution was centrifuged at 13000 rpm for 10 min at 4°C, and 10 μL of the solution was injected into LC-MS/MS. Quantification was performed by comparison with the standard curve obtained from pure nucleoside standards. The ratio of m^6^A to A in polyadenylated RNAs was calculated based on the calculated concentrations.

### Statistical Analysis

Results are presented as mean ± SEM. The m^6^A level and qRT-PCR quantification of target genes between women with and without adenomyosis were analyzed in unpaired Student’s *t*-test with SPSS software (IBM, New York, NY, United States). Statistical significance is shown as ^∗^*P* < 0.05, ^∗∗^*P* < 0.01, or ^∗∗∗^*P* < 0.001.

## Results

### Endometrium

#### m^6^A RNA Methylation Regulators Are Decreased in Endometrium of Women With Adenomyosis

**FIGURE 1:**
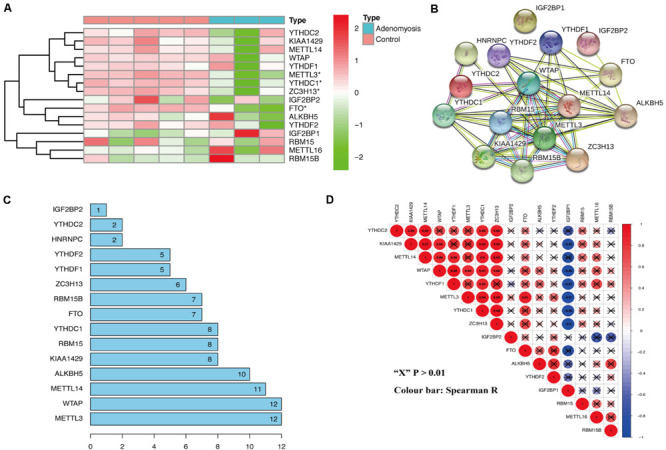
The expression and interaction of m^6^A RNA methylation regulators in the eutopic endometrium of women with and without adenomyosis. **(A)** The expression levels of sixteen m^6^A RNA methylation regulators in the eutopic endometrium. **(B)** The m^6^A modification-related interactions among the 16 m^6^A RNA methylation regulators. **(C)** The interaction counts of 16 m^6^A RNA methylation regulators. **(D)** Spearman correlation analysis of the 16 m^6^A modification regulators.

From the analysis of the gene expression profile of GSE78851, we found that *METTL3*, *ZC3H13*, *FTO*, and *YTHDC1* were all significantly decreased in the eutopic endometrium of adenomyosis patients versus controls (Figure 1A). METTL3 is a “writer,” and ZC3H13 aids in the RNA methylation process; while FTO belongs to the “erasers,” and YTHDC1 is a “reader” (Wu et al., 2020). Thus, we pursued whether m^6^A levels are regulated by the above-mentioned factors in cases versus controls. Specifically, we examined m^6^A modification-related interactions and correlations among the 16 m^6^A RNA methylation regulators. STRING database analysis suggested that *METTL3* and *WTAP* are “hub” genes of the m^6^A RNA methylation regulators, as both interact with 12 m^6^A RNA methylation regulators (interaction score ≥ 0.7) (Figures 1B,C). Moreover, the expression of *METTL3* was also significantly correlated to expression of all other differentially expressed m^6^A RNA methylation regulators in endometrium of cases versus controls, including *YTHDC1*, *FTO*, and *ZC3H13* (Figure 1D, Spearman R) without any change in the expression of *WATP*. Therefore, *METTL3* is a prime candidate as the “hub” gene of m^6^A RNA methylation regulators involved in endometrial dysfunction in the setting of adenomyosis.

#### DEGs Associated With Endometrium of Adenomyosis Women and Function Analyses

**FIGURE 2:**
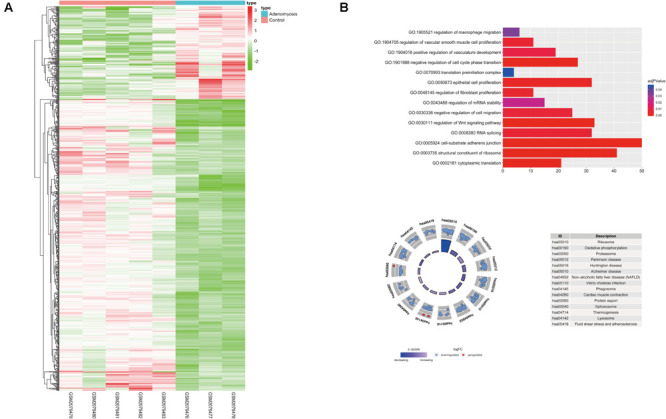
The expression, functional enrichment of DEGs in the eutopic endometrium of women with and without adenomyosis. **(A)** The differentially expressed transcripts in the eutopic endometrium of adenomyosis patients versus controls (3 adenomyosis patients and 5 controls). **(B)** Functional annotation of the DEGs of eutopic endometrium using GO terms of biological processes (upper) and KEGG pathway (lower).

Using “limma” package, we analyzed GSE78851 and found 791 transcripts differentially expressed (191 up regulated and 600 down regulated) in endometrium of cases versus controls (Figure 2A). Biological functions of these DEGs were identified using GO and KEGG enrichment. With GO, enrichment was noted in “epithelial cell proliferation,” “regulation of fibroblast proliferation,” “regulation of cell migration,” “cell-substrate adherens junction,” “regulation of Wnt signaling pathway” and other biological processes were highly enriched in DEGs [FDR (adjust *P*) < 0.05]. In addition, other significantly enriched biological processes were related to m^6^A function, including “RNA splicing,” “regulation of mRNA stability,” “translation preinitiation complex” and “structural constituent of ribosome” (Figure 2B).

#### WGCNA and Correlation Analysis to Identify Co-expression and Possible Target Genes of m^6^A RNA Methylation Regulators in Endometrium of Women With Adenomyosis

**FIGURE 3:**
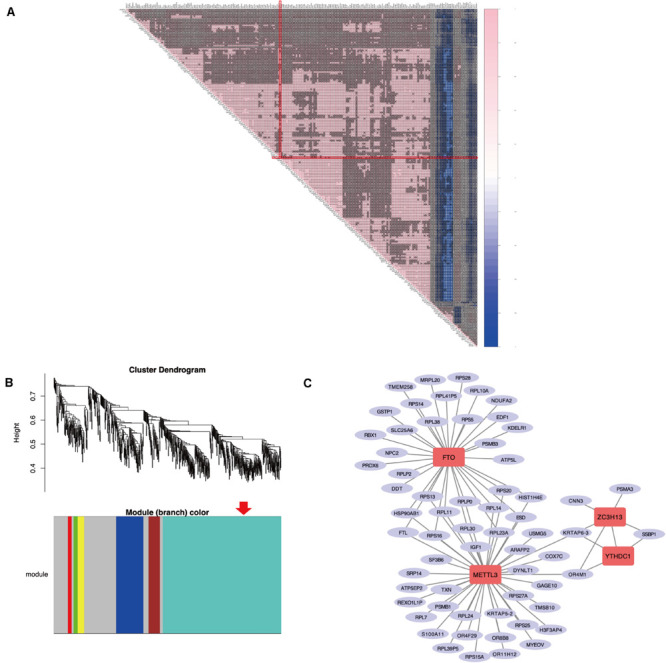
The correlation analysis and WGCNA of DEGs in the eutopic endometrium. **(A)** The Spearman correlation analysis of the m^6^A modification regulators and the DEGs enriched in the GO terms that related to eutopic endometrium dysfunction of adenomyosis. “X” *p* > 0.001, Red line clarified that correlation between METTL3 and other DEGs. **(B)** WGCNA of DEGs in the eutopic endometrium of women with adenomyosis versus without. Red arrow revealed the module that the m^6^A RNA methylation regulators belong to. **(C)** The co-expressed genes of four differential expressed m^6^A RNA methylation regulators using WGCNA analysis (threshold = 0.8).

As previously described, *METTL3* is considered as the “hub” m^6^A RNA methylation regulator. Accordingly, further exploration of the correlation between the 217 DEGs from these highly enriched GO terms and the expression of *METTL3* was performed to clarify a possible role of m^6^A RNA methylation regulators in the dysfunction of endometrium of women with adenomyosis (Figure 3A). We found 67 genes were significantly correlated to the expression of *METTL3*. Nine of these were involved in “epithelial cell proliferation,” 2 were involved in “negative regulation of cell migration” and 2 contributed to “negative regulation of cell cycle phase transition.”

WGCNA was used to further identify target genes of differentially expressed m^6^A RNA methylation regulators in endometrium of cases versus controls. Firstly, we found all differentially expressed m^6^A RNA methylation regulators (METTL3, FTO, ZC3H13, and YTHDC1) belong to the “turquoise” module (Figure 3B), demonstrating their close relationship to each other. Secondly, genes that were significantly co-expressed or correlated to the four differentially expressed m^6^A RNA methylation regulators were identified, and the network is shown in Figure 3C. Combining the Spearman correlation between *METTL3* and DEGs and WGCNA results, a total of 19 co-expressed genes were found, which may be target genes of m^6^A RNA methylation regulators in eutopic endometrium of cases versus controls (threshold = 0.8) (Table 1).

**TABLE 1:** The potential target genes of differential expressed m^6^A RNA methylation regulators in the eutopic endometrium.

**Gene name**	**GO terms**
CNN3	Cell-substrate adherens junction
RPS14	Cell-substrate adherens junction, structural constituent of ribosome
RPS16	Cell-substrate adherens junction, structural constituent of ribosome
RPS25	Cell-substrate adherens junction, structural constituent of ribosome
RPS27A	cell-substrate adherens junction, structural constituent of ribosome
RPL11	Cytoplasmic translation
IGF1	Epithelial cell proliferation
DDT	negative regulation of cell migration
PSMB1	Regulation of mRNA stability
RBX1	Regulation of Wnt signaling pathway
SF3B6	RNA splicing
RPL14	Structural constituent of ribosome
RPL23A	Structural constituent of ribosome
RPL24	Structural constituent of ribosome
RPL30	Structural constituent of ribosome
RPL38	Structural constituent of ribosome
RPL39P5	Structural constituent of ribosome
RPL7	Structural constituent of ribosome
RPLP0	Structural constituent of ribosome

#### Validation of the Total m^6^A Level, m^6^A RNA Methylation Regulators and Possible Target Genes in Endometrium of Women With Adenomyosis

**FIGURE 4:**
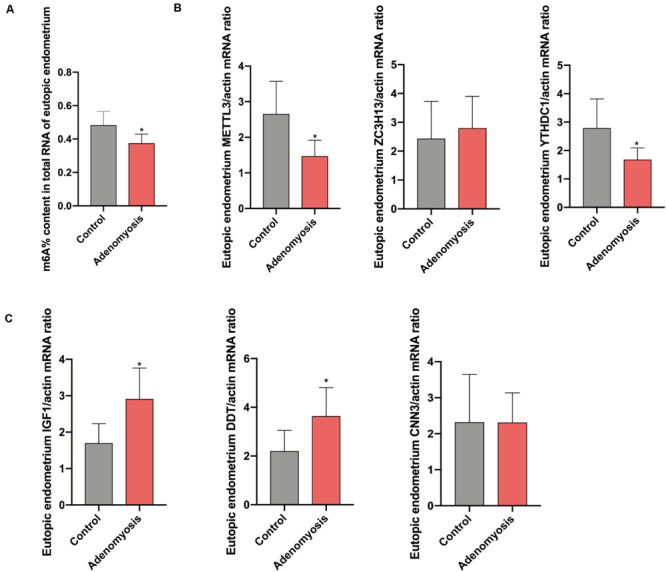
The total m^6^A level and qRT-PCR validation of bioinformatics data in eutopic endometrium of women with and without adenomyosis. **(A)** m^6^A% content in total RNA in the eutopic endometrium of women with and without adenomyosis. **(B)** The expression of *METTL3*, *ZC3H13*, *YTHDC1* mRNA level in the eutopic endometrium. **(C)** The expression of *IGF1, DDT*, and *CNN3* mRNA level in the eutopic endometrium. *n* = 6 for each group. *p < 0.05.

After combing the results from the bioinformatics analyses, m^6^A levels and *METTL3* were considered as likely functional regulators in endometrium of women with adenomyosis. To validate this hypothesis, we studied total m^6^A levels and the relative expression of m^6^A RNA methylation regulators in endometrium from a cohort of women with adenomyosis (*n* = 6) and controls (*n* = 6) – all in the proliferative phase of the menstrual cycle. The percentage of m^6^A content in total RNA of endometrium was significantly reduced (Figure 4A), while a trend for lower amounts of m^6^A was detected in polyadenylated RNA of cases versus controls (Supplementary Figure 1A), similar to endometrial cancer (Liu et al., 2018). Moreover, *METTL3*, and *YTHDC1* mRNA were also decreased (Figure 4B). We also detected METTL3 protein in endometrium via IHC. We found high expression of METTL3 protein both in the glands and stroma of control endometrium (Supplementary Figure 2A). The protein level of METTL3 was significantly decreased in the endometrium of adenomyosis patients when compared with control (*p* = 0.034), consistent with its mRNA level.

Furthermore, we verified the potential target genes of differentially expressed m^6^A RNA methylation regulators identified above. Messenger RNA for *insulin−like growth factor−1 (IGF1)*, the key regulator of the epithelial proliferation and the AKT pathway (Stitt et al., 2004; Merritt et al., 2016), was significantly increased in cases versus controls. Furthermore, *D-dopachrome tautomerase (DDT)*, works as a gene responsible for cell migration and cell proliferation (Merk et al., 2012), were also significantly highly expressed in cases versus controls, without any change in *Calponin 3 (CNN3)* (Figure 4C).

### Myometrium

#### Expression of m^6^A RNA Methylation Regulators and Functional Analysis of DEGs in Myometrium of Women With Adenomyosis

**FIGURE 5:**
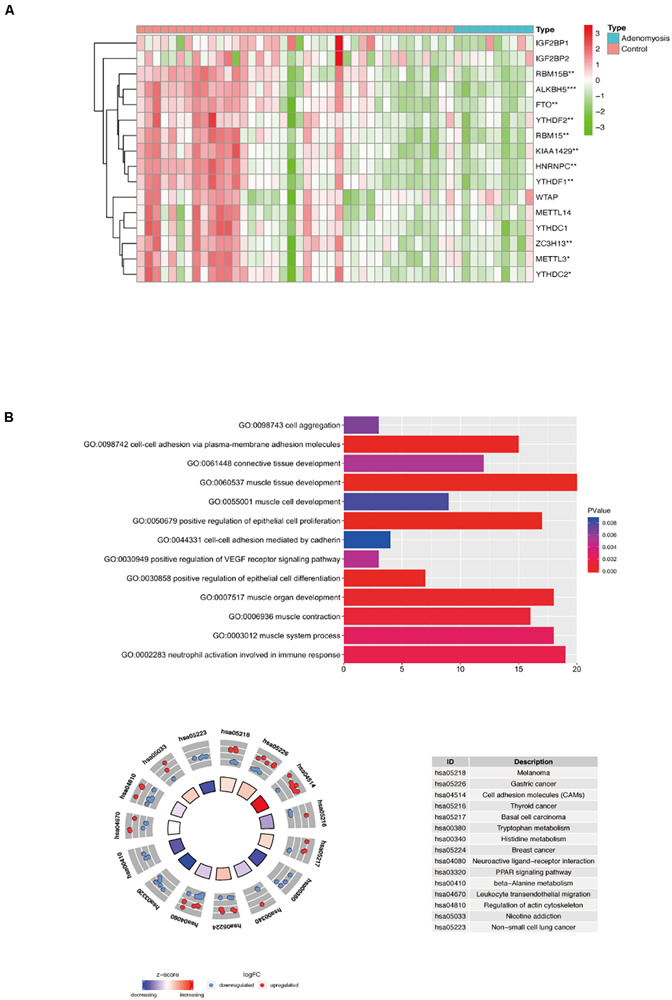
The expression of m^6^A RNA methylation regulators and the functional enrichment of DEGs in the myometrium of women with and without adenomyosis. **(A)** The expression levels of sixteen m^6^A RNA methylation regulators in the myometrium (10 women with adenomyosis and 40 without adenomyosis). **(B)** Functional enrichment of the DEGs of myometrium using GO terms of biological processes (upper) and KEGG pathway (lower).

Gene expression profiles of myometrium of women with and without adenomyosis were mined from GSE7307. Gene expression of the myometrium of adenomyosis group (*n* = 10) was compared to the control group (*n* = 40) comprised of myometrial samples without adenomyosis, endometriosis and/or cancer. The comparison identified 563 DEGs, of which 278 genes were down regulated, and 285 genes were upregulated from cases versus controls. Expression of 16 m^6^A RNA methylation regulators is shown in Figure 5A, and 11 of them were significantly differentially expressed in the myometrium of adenomyosis patients including *RBM15/15B*, *ALKBH5*, *FTO*, *YTHDF1/2*, *KIAA1429*, *HNRNPC*, *METTL3*, *ZC3H13*, and *YTHDC2* (Figure 5A).

Functional analysis revealed that “muscle tissue development,” “cell-cell adhesion via plasma-membrane adhesion molecules,” “positive regulation of epithelial cell differentiation,” “muscle contraction,” “neutrophil activation involved in immune response,” “connective tissue development” and other biological processes were highly enriched in DEGs in myometrium of cases versus controls (Figure 5B).

#### WGCNA and Correlation Analysis to Identify Potential Target Genes of m^6^A RNA Methylation Regulators in the Myometrium

**FIGURE 6:**
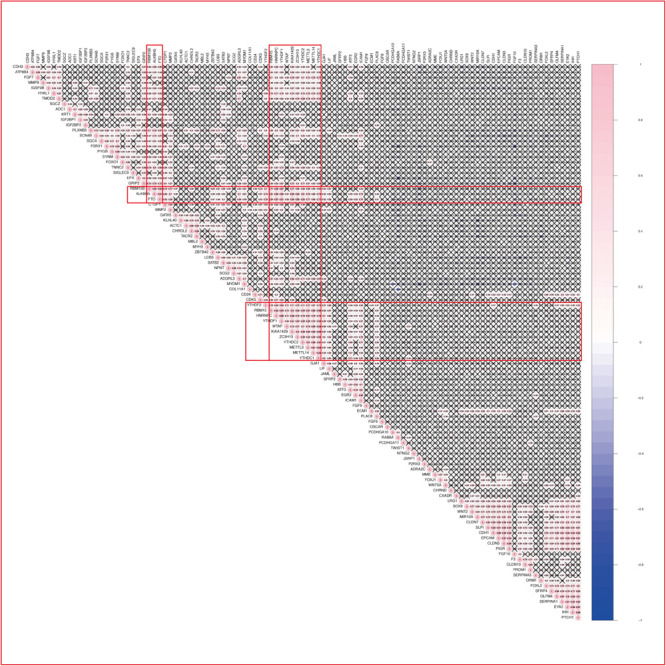
The Spearman correlation between m^6^A RNA methylation regulators and the DEGs of myometrium enriched in the GO terms that related to myometrium dysfunction of adenomyosis patients. “X” *p* > 0.001, Red line clarified that correlation between the 13 m^6^A regulation “cluster” and other DEGs.

Different from endometrium, 13 of 16 m^6^A RNA methylation regulators in myometrium were significantly correlated to each other, and most of them were down regulated in adenomyosis patients using Spearman correlation (Figures 5A, 6). Additionally, the 13 m^6^A RNA methylation regulators were all attributed to the “blue” module using WGCNA (Supplementary Figure 3). Thus, the 13 m^6^A RNA methylation regulators (*RBM15/15B*, *YTHDF1*, *WTAP*, *KIAA1429*, *ZC3H13*, *YTHDC2*, *METTL3*, *METTL14*, *YTHDC1*, *ALKBH5*, and *FTO*) were identified as a “cluster” of m^6^A methylation regulators in the myometrium of women.

We analyzed the correlation between the 107 DEGs in these highly enriched biological processes in myometrium of women with adenomyosis and controls and m^6^A regulator “cluster” to clarify potential mechanisms underlying regulation of m^6^A RNA methylation regulators in the setting of disease. Genes that significantly correlated to the expression of the “m^6^A regulator cluster” were identified as potential target genes of m^6^A RNA methylation regulators in the myometrium. Fifteen genes were identified, 4 of which were involved in “muscle contraction”, 3 with “cell-cell adhesion” and 2 genes were involved in “neutrophil activation involved in immune response” (Table 2; Figure 6).

**TABLE 2:** The DEGs from high enriched GO terms that are significantly correlated to m^6^A RNA methylation regulators cluster in the myometrium.

**Gene name**	**GO terms**
CDH3	Cell–cell adhesion mediated by cadherin
IGSF9B	Cell–cell adhesion via plasma-membrane adhesion molecules
ADGRL3	Cell–cell adhesion via plasma-membrane adhesion molecules
SCN4B	Muscle contraction
TNNC2	Muscle contraction
TMOD2	Muscle contraction
GRIP2	Muscle contraction
ATF3	Muscle organ development
EGR2	Muscle organ development
ATP8B4	Neutrophil activation involved in immune response
PLAC8	Neutrophil activation involved in immune response
LIF	Positive regulation of epithelial cell differentiation
FGF7	Positive regulation of epithelial cell proliferation
HYAL1	Positive regulation of epithelial cell proliferation
PLXNB3	Positive regulation of epithelial cell proliferation

#### Validation of the Total m^6^A Level, m^6^A RNA Methylation Regulators and Possible Downstream Factors in the Myometrium of Women With Adenomyosis

**FIGURE 7:**
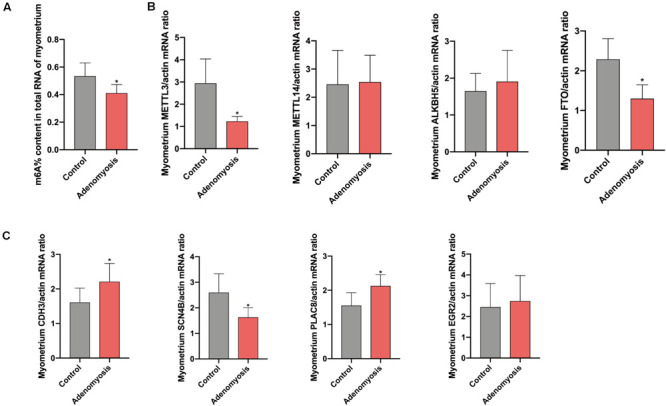
The total m^6^A level and qRT-PCR validation of bioinformation data in myometrium of women with and without adenomyosis. **(A)** m^6^A% content in total RNA in the myometrium of adenomyosis and control patients. **(B)** The expression of *METTL3*, *METTL14*, *ALKBH5*, *FTO* mRNA level in the myometrium. **(C)** The expression of *CDH3, SCN4B, PLAC8*, and *EGR2* mRNA level in the myometrium. *n* = 6 for each group. *p < 0.05.

Equivalent to the eutopic endometrium, the m^6^A% content in total RNA of myometrium was also significantly reduced (Figure 7A) while a decreased tendency of m^6^A% was detected in polyadenylated RNA of women with adenomyosis compared with controls (Supplementary Figure 1B). In addition, expression of *METTL3* and *FTO* mRNA was decreased without changes in *METTL14* and *ALKBH5* (Figure 7B). The protein level of METTL3 appeared lower in myometrium of women with adenomyosis compared with controls (*p* = 0.198) (Supplementary Figure 2B). Expression of identified genes that were significantly correlated with the m^6^A regulator “cluster” in the myometrium was also verified. As a key molecule in cell-cell adhesion and EMT (Sousa et al., 2019), *cadherin 3 (CDH3)* mRNA was significantly increased in adenomyosis patients. Additionally, *sodium channel*β*-subunit 4 (SCN4B)* mRNA was decreased, suggesting possible regulation of m^6^A RNA methylation regulators to cell adhesion in adenomyosis. Finally, *placenta-specific protein 8 (PLAC8)* mRNA was also increased in the myometrium of adenomyosis patients, indicating the role of immune response in the myometrium of women with adenomyosis (Johnson et al., 2012; Figure 7C).

## Discussion

### Both Endometrium and Myometrium Dysfunction Contribute to the Pathogenesis of Adenomyosis

Adenomyosis is a disease with unknown pathogenesis, and diagnostics rely on the utilization of imaging techniques based on differences in the appearance of smooth muscle, particularly the inner myometrium (Kissler et al., 2008). One hypothesis of the pathogenesis of adenomyosis is that the adenomyosis lesions originate from invaginating endometrium basalis, due to the similarities between these tissues. Thus, the disruption of the normal boundary (inner myometrium) may result in invasion of endometrial cells into the myometrium, inducing myocyte hypertrophy (Vercellini et al., 2006). At the same time, *in vitro* studies have demonstrated that myocytes from adenomyosis enhance invasion of endometrial stromal cells, compared to normal myocytes. Moreover, some studies have reported misexpression of estrogen receptor (ER) and progesterone receptor (PR) in the inner myometrium, including increased ER-β and decreased PR-A and PR-B (Mehasseb et al., 2011b). While myometrial dysfunction in the pathogenesis of adenomyosis has been proposed (Mehasseb et al., 2010b), other studies demonstrate that disruption of the myometrium does not necessarily result in adenomyosis (Mehasseb et al., 2010a). With regard to the endometrium, there is increased invasiveness of the E-cadherin negative epithelial cells (Gaetje et al., 1997; Benagiano and Brosens, 2012; Brosens et al., 2012) and abnormal estrogen. Thus, adenomyosis appears to be a disease of both the myometrial and endometrial compartments, although further research is needed to understand mechanisms contributing to the pathogenesis and pathophysiology of this disorder.

### m^6^A RNA Methylation Regulators Are Involved in Eutopic Endometrium Dysfunction of Women With Adenomyosis

RNA methylation, especially m^6^A, contributes to biological processes such as cell proliferation, immunology (Winkler et al., 2019; Zhang et al., 2019), tumorigenesis (Deng et al., 2018) and tissue development (Klungland and Dahl, 2014; Wu et al., 2018; Heck and Wilusz, 2019), all of which may be involved in the pathogenesis of adenomyosis. Moreover, reduced *METTL3* and m^6^A level have been detected in endometrial cancer, which shares some characteristics with adenomyosis.

*METTL3* expression was significantly decreased in the endometrium of adenomyosis patients with reduced total m^6^A levels, similar to those in endometrial cancer (Liu et al., 2018). As a result, reduced m^6^A may contribute to the endometrial dysfunction in the setting of adenomyosis via different functional pathways. We found that DEGs were involved in epithelial cell proliferation, vasculature development, cell migration and macrophage migration, processes consistent with recent RNA-seq data of the endometrium in women with adenomyosis (Xiang et al., 2019). Moreover, Benagiano and Brosens have also suggested that some of enriched biological processes (e.g., increase angiogenesis, proliferation of endometrium and Wnt signaling pathway) are involved in the pathogenesis of adenomyosis (Benagiano and Brosens, 2012). We further investigated the correlation and co-expression among the DEGs in these biological processes and *METTL3*, the “hub” gene of m^6^A RNA methylation regulators in the endometrium, suggesting the possible target genes of *METTL3* and m^6^A regulation.

IGF1 is an effective growth factor in disease progression and plays an important role in cellular growth, proliferation, invasion, and angiogenesis of several tissues. Our study revealed that *IGF1* was differentially expressed in the microarray analysis and was significantly correlated with expression of *METTL3* in the endometrium of adenomyosis patients. Previous study demonstrated that the decreased METTL3 and m^6^A RNA methylation level can promote the cell proliferation through the AKT pathway in the endometrium, while IGF1 contributes to the regulation of AKT (Liu et al., 2018). Thus, combining the bioinformatic analyses herein with data from the literature, we propose that *METTL3* regulates m^6^A and contributes to increased expression of *IGF1*, which further promotes cell proliferation and invasion of endometrial cells into the myometrium via AKT pathway. However, the mechanism underlying the regulation of METTL3 and m^6^A to IGF1 and AKT pathway still need to be further investigated.

EMT, induced in the basal endometrium by high levels of estrogen, resulted in invagination of endometrium into myometrium, thereby playing an important role in the pathogenesis of adenomyosis (Hu et al., 2020). Herein, in our data analysis we found the decreased expression of *cadherin 1*(E-cadherin), a marker of the epithelial cell and EMT. Wnt pathway activation induces EMT in several tissues (Teeuwssen and Fodde, 2019); we found that *WNT5A* mRNA was differentially expressed in the endometrium of adenomyosis patients. *WNT5A* may contribute to the pathogenesis of adenomyosis through proliferation of epithelial and fibroblast cells and regulation of vasculature development. However, expression of *WNT5A* was not correlated to *METTL3* in endometrium, suggesting it is not under the control of m^6^A RNA methylation regulators. Beside Wnt signaling, previous studies demonstrated that METTL3 and m^6^A levels contribute to EMT in lung cancer (Wanna-Udom et al., 2020). IGF1 upregulates components of the Wnt signaling pathway and promotes EMT, and it is co-expressed with METTL3 (Verras and Sun, 2005). Thus, increased *IGF1* in eutopic endometrium may mediate the regulation of METTL3 and contribute to the endometrial dysfunction in adenomyosis women through EMT.

Besides of EMT, multipotent stem cells that travel from the endometrium to the myometrium and subsequently differentiate into lineage epithelial and/or stromal fibroblasts provide another possibility for adenomyosis pathogenesis. METTL3 and m^6^A can also contribute to the differentiation of stem cells in different tissues, indicating another possible role of m^6^A and METTL3 in adenomyosis (Lee et al., 2019).

DDT is the a member of the macrophage migration inhibitory factor (MIF) protein superfamily (Merk et al., 2012) and is involved in regulation of cell migration and other biological processes in various tumors. Previous studies found that DDT and MIF are involved in proliferation, migration, and invasion of cervical cancer (Wang et al., 2017). Moreover, MIF contributes to development of endometriosis, and MIF expression is increased in the endometrium of adenomyosis women (Rakhila et al., 2014). In our data analysis results, we also found MIF to be significantly differentially expressed in the endometrium of women with adenomyosis. However, its correlation to *METTL3* and other m^6^A RNA methylation regulators was not significant. Thus, MIF may be involved in endometrial dysfunction of women with adenomyosis, but not under the regulation of m^6^A RNA methylation regulators. DDT is significantly co-expressed with m^6^A RNA methylation regulators and binds to MIF cell surface receptor, inducing similar cell signaling and effector functions. Thus, the increased *DDT* mRNA in the endometrium of adenomyosis patients could mediate regulation of m^6^A RNA methylation regulators relevant to cell migration of endometrium in the setting of adenomyosis.

### m^6^A RNA Methylation Regulators Play Roles in the Myometrium Dysfunction of Women With Adenomyosis

Our study also revealed decreased m^6^A content and 11 differentially expressed m^6^A RNA methylation regulators in myometrium of adenomyosis patients, providing a possible mechanism for myometrial dysfunction of adenomyosis. Moreover, downstream factors of m^6^A regulation may participate.

While immune activation is mainly observed in endometrium of women with adenomyosis, e.g., increased endometrial macrophages and autoantibodies such as anti-phosphatidylinositol IgG, anti-phosphatidylglycerol IgG (Ota et al., 1998), immune dysregulation may also occur in the myometrium of affected women. m^6^A has been shown to be involved in immune regulation (Wang et al., 2019), and our functional enrichment analysis of DEGs in myometrium herein highlights neutrophil activation in cases versus controls. Additionally, PLAC8, a multi-faceted protein involved in various cellular physical processes (such as the regulation of immunity, cell differentiation and apoptosis) (Li et al., 2014), was also increased in the myometrium in adenomyosis patients and was closely related to m^6^A RNA methylation regulator “cluster.” Thus, the myometrium has processes in place for immune cell response in the setting of adenomyosis, associated with m^6^A RNA methylation regulators.

SCN4B is the β-subunit of voltage-gated sodium channels (VGSCs), required for generation of action potentials in excitable cells, and it also functions in cell–cell adhesion (Shimizu et al., 2017). Expression of *SCN4B* has been detected in the longitudinal smooth muscle layer of rat myometrium (Seda et al., 2007), suggesting its role in myometrial homeostasis and contractility. Functional enrichment analysis of myometrial DEGs in our study identified SCN4B associated with “muscle contraction” and was also significantly correlated with expression of the m^6^A regulator “cluster.” Moreover, expression of *SCN4B* mRNA was decreased in myometrium of adenomyosis patients, as were *METTL3* and *FTO*. Thus, m^6^A RNA methylation regulators may regulate cell adhesion through *SCN4B*, further contributing to adenomyosis development.

In the myometrium, the GO enrichment of DEGs revealed that *WNT5A* was decreased and was involved in “the connective tissue development,” “muscle development” and other processes. However, it was also not correlated to the m^6^A RNA methylation regulators “cluster” herein. Thus, *WNT5A* may contribute to the pathogenesis of adenomyosis but is not regulated through the regulation of m^6^A RNA methylation regulators.

### Strengths and Limitations

The strengths of this study include mining publicly available databases with abundant gene expression on human endometrium and myometrium separately from women with and without adenomyosis. Also, the endometrial and myometrial specimens used for validation were obtained using standard operating procedures (SOPs) from the UCSF NIH Human Endometrial Tissue and DNA Bank with well annotated clinical data in our RedCap Database (Sheldon et al., 2011^3^). Moreover, our study is the first to propose possible involvement of RNA methylation in the pathogenesis of adenomyosis. It thus provides a novel paradigm needing subsequent mechanistic validation. It also potentially opens new avenues for novel targeted therapeutic approaches for symptoms associated with this disorder.

The limitations of our study were that the roles of the m^6^A RNA methylation regulators and their downstream factors in adenomyosis was deduced from gene expression profile analysis. Mechanisms underlying a role for m^6^A RNA methylation regulation still needs to be demonstrated through animal and *in vitro* experiments. In addition, although we did the validation using clinical samples, the sample size was limited, and the protein levels of target genes still needed to be detected. Other limitations include: the type of adenomyosis lesions (diffuse, adenomyoma, or cystic), clinical data, and cycle phase of subjects of the myometrium samples used in the data reported in the GEO database are not known. Notably, our validation approach used myometrium solely from the proliferative phase of the cycle, and previous studies have demonstrated expression of some genes in myometrium in adenomyosis patients do not vary with cycle phase (Mehasseb et al., 2011a), giving some mitigation to this limitation.

## Summary and Conclusion

Herein, we investigated gene expression and the interactome of m^6^A RNA methylation regulators and total m^6^A levels in adenomyosis patients. Decreased *METTL3* and total m^6^A levels in endometrium of adenomyosis patients may contribute to cell proliferation and invasion through *IGF1* and *DDT*. The RNA methylation levels of specific and target genes such as *IGF1*, *DDT, PLAC8*, and *SCN4B* remain to be investigated using methods such as methylated RNA immunoprecipitation sequencing (MeRIP-seq) and MeRIP-qPCR. Furthermore, in the myometrium, m^6^A RNA methylation regulators work as a cluster and play roles in cell adhesion, muscle contraction and immune response. In conclusion, m^6^A RNA methylation regulators may be involved the pathogenesis of adenomyosis through aberrant expression and actions in both the uterine endometrium and myometrium.

## Data Availability Statement

The two public gene expression datasets GSE78851 and GSE7303 can be downloaded from the GEO database (https://www.ncbi.nlm.nih.gov/geo/). All other data presented in this study are included in the article/Supplementary Material.

## Ethics Statement

The studies involving human participants were reviewed and approved by the Committee on Human Research (CHR) at UCSF. The patients/participants provided their written informed consent to participate in this study.

## Author Contributions

JZ, SL, JO-A, and LG contributed to the design of the experiments, collection of samples, acquisition of data, analysis and interpretation of data. JZ, SL, YD, and Z-JC analyzed the data and made the figures. JZ, SS, and LG finished drafting the manuscript or revising it critically for important intellectual content. LG is responsible for the final approval of the version to be published. All authors contributed to the article and approved the submitted version.

## Conflict of Interest

The authors declare that the research was conducted in the absence of any commercial or financial relationships that could be construed as a potential conflict of interest.

## Footnotes

1 https://www.ncbi.nlm.nih.gov/geo/

2 http://www.string-db.org/

3 https://redcap.ucsf.edu/redcap_v9.5.14

## Supplementary Material

FIGURE S1 m^6^A% content in the polyadenylated RNA of eutopic endometrium and myometrium of women with and without adenomyosis. **(A)** Eutopic endometrium; **(B)** Myometrium. *n* = 3 samples were analyzed in each group.

FIGURE S2 The IHC of METTL3 in the endometrium and myometrium of women with and without adenomyosis. **(A)** The endometrium of women with (right panel) and without adenomyosis (left panel). **(B)** the myometrium of women with (right panel) and without adenomyosis (left panel). Staining was assessed using Image J (*n* = 3 for each group) and scored on a scale of 0 (negative) to 3+ (high positive). Scale bars is 50 μm. The *p*-value was determined by a χ^2^-test.

FIGURE S3 The WGCNA of DEGs in the myometrium of women with and without adenomyosis. Red arrow revealed the module that the differential expressed m^6^A RNA methylation regulators belongs to.

## References

[B1] Arnold L. L. Meck J. M. Simon J. A. (1995). Adenomyosis: evidence for genetic cause. *Am. J. Med. Genet.* 55 505–506. 10.1002/ajmg.1320550423 7762596

[B2] Benagiano G. Brosens I. (2012). The endometrium in adenomyosis. *Womens Health* 8 301–312. 10.2217/whe.12.8 22554177

[B3] Benagiano G. Habiba M. Brosens I. (2012). The pathophysiology of uterine adenomyosis: an update. *Fertil. Steril.* 98 572–579. 10.1016/j.fertnstert.2012.06.044 22819188

[B4] Bird C. C. Mcelin T. W. Manalo-Estrella P. (1972). The elusive adenomyosis of the uterus–revisited. *Am. J. Obstet. Gynecol.* 112 583–593. 10.1016/0002-9378(72)90781-8 5059589

[B5] Brosens I. Brosens J. J. Benagiano G. (2012). The eutopic endometrium in endometriosis: are the changes of clinical significance? *Reprod. Biomed. Online* 24 496–502. 10.1016/j.rbmo.2012.01.022 22417665

[B6] Curtis K. M. Hillis S. D. Marchbanks P. A. Peterson H. B. (2002). Disruption of the endometrial-myometrial border during pregnancy as a risk factor for adenomyosis. *Am. J. Obstet. Gynecol.* 187 543–544. 10.1067/mob.2002.124285 12237624

[B7] Deng X. Su R. Feng X. Wei M. Chen J. (2018). Role of N(6)-methyladenosine modification in cancer. *Curr. Opin. Genet. Dev.* 48 1–7. 10.1016/j.gde.2017.10.005 29040886 PMC5869081

[B8] Gaetje R. Kotzian S. Herrmann G. Baumann R. Starzinski-Powitz A. (1997). Nonmalignant epithelial cells, potentially invasive in human endometriosis, lack the tumor suppressor molecule E-cadherin. *Am. J. Pathol.* 150 461–467. 9033262 PMC1858282

[B9] Gargett C. E. Schwab K. E. Deane J. A. (2016). Endometrial stem/progenitor cells: the first 10 years. *Hum. Reprod. Update* 22 137–163. 26552890 10.1093/humupd/dmv051 PMC4755439

[B10] Gu S. Sun D. Dai H. Zhang Z. (2018). N(6)-methyladenosine mediates the cellular proliferation and apoptosis via microRNAs in arsenite-transformed cells. *Toxicol. Lett.* 292 1–11. 10.1016/j.toxlet.2018.04.018 29680375

[B11] Heck A. M. Wilusz C. J. (2019). Small changes, big implications: the impact of m(6)A RNA methylation on gene expression in pluripotency and development. *Biochim. Biophys. Acta Gene Regul. Mech.* 1862:194402. 10.1016/j.bbagrm.2019.07.003 31325527 PMC6742438

[B12] Herndon C. N. Aghajanova L. Balayan S. Erikson D. Barragan F. Goldfien G. (2016). Global transcriptome abnormalities of the eutopic endometrium from women with adenomyosis. *Reprod. Sci.* 23 1289–1303. 10.1177/1933719116650758 27233751 PMC6344825

[B13] Hu R. Peng G. Q. Ban D. Y. Zhang C. Zhang X. Q. Li Y. P. (2020). High-expression of neuropilin 1 correlates to estrogen-induced epithelial-mesenchymal transition of endometrial cells in adenomyosis. *Reprod. Sci.* 27 395–403. 10.1007/s43032-019-00035-2 32046395

[B14] Inoue S. Hirota Y. Ueno T. Fukui Y. Yoshida E. Hayashi T. (2019). Uterine adenomyosis is an oligoclonal disorder associated with KRAS mutations. *Nat. Commun.* 10:5785. 10.1038/s41467-019-13708-y PMC6923389 31857578

[B15] Johnson R. M. Kerr M. S. Slaven J. E. (2012). Plac8-dependent and inducible NO synthase-dependent mechanisms clear *Chlamydia muridarum* infections from the genital tract. *J. Immunol.* 188 1896–1904. 10.4049/jimmunol.1102764 22238459 PMC3303601

[B16] Kissler S. Zangos S. Kohl J. Wiegratz I. Rody A. Gatje R. (2008). Duration of dysmenorrhoea and extent of adenomyosis visualised by magnetic resonance imaging. *Eur. J. Obstet. Gynecol. Reprod. Biol.* 137 204–209. 10.1016/j.ejogrb.2007.01.015 17397990

[B17] Klungland A. Dahl J. A. (2014). Dynamic RNA modifications in disease. *Curr. Opin. Genet. Dev.* 26 47–52. 10.1016/j.gde.2014.05.006 25005745

[B18] Langfelder P. Horvath S. (2008). WGCNA: an R package for weighted correlation network analysis. *BMC Bioinformatics* 9:559. 10.1186/1471-2105-9-559 19114008 PMC2631488

[B19] Lee H. Bao S. Qian Y. Geula S. Leslie J. Zhang C. (2019). Stage-specific requirement for Mettl3-dependent m(6)A mRNA methylation during haematopoietic stem cell differentiation. *Nat. Cell. Biol.* 21 700–709. 10.1038/s41556-019-0318-1 31061465 PMC6556891

[B20] Li C. Ma H. Wang Y. Cao Z. Graves-Deal R. Powell A. E. (2014). Excess PLAC8 promotes an unconventional ERK2-dependent EMT in colon cancer. *J. Clin. Invest.* 124 2172–2187. 10.1172/jci71103 24691442 PMC4001536

[B21] Li J. Chen F. Peng Y. Lv Z. Lin X. Chen Z. (2020). N6-methyladenosine regulates the expression and secretion of tgfbeta1 to affect the epithelial-mesenchymal transition of cancer cells. *Cells* 9:296. 10.3390/cells9020296 31991845 PMC7072279

[B22] Li J. Yanyan M. Mu L. Chen X. Zheng W. (2019). The expression of Bcl-2 in adenomyosis and its effect on proliferation, migration, and apoptosis of endometrial stromal cells. *Pathol. Res. Pract.* 215:152477. 10.1016/j.prp.2019.152477 31174926

[B23] Li J. J. Chung J. P. W. Wang S. Li T. C. Duan H. (2018). The investigation and management of adenomyosis in women who wish to improve or preserve fertility. *Biomed. Res. Int.* 2018:6832685. 10.1155/2018/6832685 PMC5875064 29736395

[B24] Liao S. Sun H. Xu C. (2018). YTH domain: a family of N(6)-methyladenosine (m(6)A) readers. *Genomics Proteomics Bioinformatics* 16 99–107. 10.1016/j.gpb.2018.04.002 29715522 PMC6112328

[B25] Liu J. Eckert M. A. Harada B. T. Liu S. M. Lu Z. Yu K. (2018). m(6)A mRNA methylation regulates AKT activity to promote the proliferation and tumorigenicity of endometrial cancer. *Nat. Cell. Biol.* 20 1074–1083. 10.1038/s41556-018-0174-4 30154548 PMC6245953

[B26] Liu J. Yue Y. Han D. Wang X. Fu Y. Zhang L. (2014). A METTL3-METTL14 complex mediates mammalian nuclear RNA N6-adenosine methylation. *Nat. Chem. Biol.* 10 93–95. 10.1038/nchembio.1432 24316715 PMC3911877

[B27] Mehasseb M. K. Bell S. C. Brown L. Pringle J. H. Habiba M. (2011a). Phenotypic characterisation of the inner and outer myometrium in normal and adenomyotic uteri. *Gynecol. Obstet. Invest.* 71 217–224. 10.1159/000318205 21160148

[B28] Mehasseb M. K. Bell S. C. Habiba M. A. (2010a). Neonatal administration of tamoxifen causes disruption of myometrial development but not adenomyosis in the C57/BL6J mouse. *Reproduction* 139 1067–1075. 10.1530/rep-09-0443 20368191

[B29] Mehasseb M. K. Panchal R. Taylor A. H. Brown L. Bell S. C. Habiba M. (2011b). Estrogen and progesterone receptor isoform distribution through the menstrual cycle in uteri with and without adenomyosis. *Fertil. Steril.* 95 2228.e1–2235.e1. 21444077 10.1016/j.fertnstert.2011.02.051

[B30] Mehasseb M. K. Taylor A. H. Pringle J. H. Bell S. C. Habiba M. (2010b). Enhanced invasion of stromal cells from adenomyosis in a three-dimensional coculture model is augmented by the presence of myocytes from affected uteri. *Fertil. Steril.* 94 2547–2551. 10.1016/j.fertnstert.2010.04.016 20537634

[B31] Merk M. Mitchell R. A. Endres S. Bucala R. (2012). D-dopachrome tautomerase (D-DT or MIF-2): doubling the MIF cytokine family. *Cytokine* 59 10–17. 10.1016/j.cyto.2012.03.014 22507380 PMC3367028

[B32] Merritt M. A. Strickler H. D. Einstein M. H. Yang H. P. Sherman M. E. Wentzensen N. (2016). Insulin/IGF and sex hormone axes in human endometrium and associations with endometrial cancer risk factors. *Cancer Causes Control* 27 737–748. 10.1007/s10552-016-0751-4 27125830 PMC4870288

[B33] Noyes R. W. Hertig A. T. Rock J. (1950). Dating the endometrial biopsy. *Fertil. Steril.* 1 3–25. 10.1016/j.fertnstert.2019.08.079 31623748

[B34] Ota H. Igarashi S. Hatazawa J. Tanaka T. (1998). Is adenomyosis an immune disease? *Hum. Reprod. Update* 4 360–367. 10.1093/humupd/4.4.360 9825851

[B35] Peer E. Rechavi G. Dominissini D. (2017). Epitranscriptomics: regulation of mRNA metabolism through modifications. *Curr. Opin. Chem. Biol.* 41 93–98. 10.1016/j.cbpa.2017.10.008 29125941

[B36] Rakhila H. Girard K. Leboeuf M. Lemyre M. Akoum A. (2014). Macrophage migration inhibitory factor is involved in ectopic endometrial tissue growth and peritoneal-endometrial tissue interaction in vivo: a plausible link to endometriosis development. *PLoS One* 9:e110434. 10.1371/journal.pone.0110434 25329068 PMC4201552

[B37] Reinhold C. Tafazoli F. Wang L. (1998). Imaging features of adenomyosis. *Hum. Reprod. Update* 4 337–349. 10.1093/humupd/4.4.337 9825849

[B38] Roddy E. Chapman J. (2017). Genomic insights in gynecologic cancer. *Curr. Probl. Cancer* 41 8–36. 10.1016/j.currproblcancer.2016.11.001 28088330

[B39] Roundtree I. A. He C. (2016). Nuclear m(6)A Reader YTHDC1 Regulates mRNA Splicing. *Trends Genet.* 32 320–321. 10.1016/j.tig.2016.03.006 27050931

[B40] Seda M. Pinto F. M. Wray S. Cintado C. G. Noheda P. Buschmann H. (2007). Functional and molecular characterization of voltage-gated sodium channels in uteri from nonpregnant rats. *Biol. Reprod.* 77 855–863. 10.1095/biolreprod.107.063016 17671266

[B41] Sheldon E. Vo K. C. Mcintire R. A. Aghajanova L. Zelenko Z. Irwin J. C. (2011). Biobanking human endometrial tissue and blood specimens: standard operating procedure and importance to reproductive biology research and diagnostic development. *Fertil. Steril.* 95 2120–2122. 21371706 10.1016/j.fertnstert.2011.01.164 PMC3080464

[B42] Shimizu H. Tosaki A. Ohsawa N. Ishizuka-Katsura Y. Shoji S. Miyazaki H. (2017). Parallel homodimer structures of the extracellular domains of the voltage-gated sodium channel beta4 subunit explain its role in cell-cell adhesion. *J. Biol. Chem.* 292 13428–13440. 10.1074/jbc.m117.786509 28655765 PMC5555201

[B43] Sousa B. Pereira J. Paredes J. (2019). The crosstalk between cell adhesion and cancer metabolism. *Int. J. Mol. Sci.* 20:1933. 10.3390/ijms20081933 31010154 PMC6515343

[B44] Stitt T. N. Drujan D. Clarke B. A. Panaro F. Timofeyva Y. Kline W. O. (2004). The IGF-1/PI3K/Akt pathway prevents expression of muscle atrophy-induced ubiquitin ligases by inhibiting FOXO transcription factors. *Mol. Cell.* 14 395–403. 10.1016/s1097-2765(04)00211-4 15125842

[B45] Teeuwssen M. Fodde R. (2019). Wnt signaling in ovarian cancer stemness, EMT, and therapy resistance. *J. Clin. Med.* 8:1658. 10.3390/jcm8101658 31614568 PMC6832489

[B46] Vercellini P. Vigano P. Somigliana E. Daguati R. Abbiati A. Fedele L. (2006). Adenomyosis: epidemiological factors. *Best Pract. Res. Clin. Obstet. Gynaecol.* 20 465–477. 10.1016/j.bpobgyn.2006.01.017 16563868

[B47] Verras M. Sun Z. (2005). Beta-catenin is involved in insulin-like growth factor 1-mediated transactivation of the androgen receptor. *Mol. Endocrinol.* 19 391–398. 10.1210/me.2004-0208 15514031

[B48] Wang H. Hu X. Huang M. Liu J. Gu Y. Ma L. (2019). Mettl3-mediated mRNA m(6)A methylation promotes dendritic cell activation. *Nat. Commun.* 10:1898. 10.1038/s41467-019-09903-6 PMC6478715 31015515

[B49] Wang Q. Wei Y. Zhang J. (2017). Combined knockdown of D-dopachrome tautomerase and migration inhibitory factor inhibits the proliferation, migration, and invasion in human cervical cancer. *Int. J. Gynecol. Cancer* 27 634–642. 10.1097/igc.0000000000000951 28338494

[B50] Wang X. Lu Z. Gomez A. Hon G. C. Yue Y. Han D. (2014). N6-methyladenosine-dependent regulation of messenger RNA stability. *Nature* 505 117–120. 10.1038/nature12730 24284625 PMC3877715

[B51] Wanna-Udom S. Terashima M. Lyu H. Ishimura A. Takino T. Sakari M. (2020). The m6A methyltransferase METTL3 contributes to Transforming Growth Factor-beta-induced epithelial-mesenchymal transition of lung cancer cells through the regulation of JUNB. *Biochem. Biophys. Res. Commun.* 524 150–155. 10.1016/j.bbrc.2020.01.042 31982139

[B52] Wen J. Lv R. Ma H. Shen H. He C. Wang J. (2018). Zc3h13 regulates nuclear RNA m(6)a methylation and mouse embryonic stem cell self-renewal. *Mol. Cell.* 69 1028.e6–1038.e6. 29547716 10.1016/j.molcel.2018.02.015 PMC5858226

[B53] Winkler R. Gillis E. Lasman L. Safra M. Geula S. Soyris C. (2019). m(6)A modification controls the innate immune response to infection by targeting type I interferons. *Nat. Immunol.* 20 173–182. 10.1038/s41590-018-0275-z 30559377

[B54] Wu J. Frazier K. Zhang J. Gan Z. Wang T. Zhong X. (2020). Emerging role of m(6) A RNA methylation in nutritional physiology and metabolism. *Obes. Rev.* 21:e12942. 10.1111/obr.12942 PMC7427634 31475777

[B55] Wu R. Liu Y. Yao Y. Zhao Y. Bi Z. Jiang Q. (2018). FTO regulates adipogenesis by controlling cell cycle progression via m(6)A-YTHDF2 dependent mechanism. *Biochim. Biophys. Acta Mol. Cell. Biol. Lipids* 1863 1323–1330. 10.1016/j.bbalip.2018.08.008 30305247

[B56] Xiang Y. Sun Y. Yang B. Yang Y. Zhang Y. Yu T. (2019). Transcriptome sequencing of adenomyosis eutopic endometrium: a new insight into its pathophysiology. *J. Cell. Mol. Med.* 23 8381–8391. 10.1111/jcmm.14718 31576674 PMC6850960

[B57] Yeh C. C. Su F. H. Tzeng C. R. Muo C. H. Wang W. C. (2018). Women with adenomyosis are at higher risks of endometrial and thyroid cancers: a population-based historical cohort study. *PLoS One* 13:e0194011. 10.1371/journal.pone.0194011 29522577 PMC5844548

[B58] Yue Y. Liu J. Cui X. Cao J. Luo G. Zhang Z. (2018). VIRMA mediates preferential m(6)A mRNA methylation in 3’UTR and near stop codon and associates with alternative polyadenylation. *Cell Discov.* 4:10. 10.1038/s41421-018-0019-0 PMC5826926 29507755

[B59] Zaccara S. Ries R. J. Jaffrey S. R. (2019). Reading, writing and erasing mRNA methylation. *Nat. Rev. Mol. Cell. Biol.* 20 608–624. 10.1038/s41580-019-0168-5 31520073

[B60] Zhang C. Fu J. Zhou Y. (2019). A review in research progress concerning m6A methylation and immunoregulation. *Front. Immunol.* 10:922. 10.3389/fimmu.2019.00922 31080453 PMC6497756

