# Supplementary figures and images for: m6A RNA Methylation Regulators Contribute to Eutopic Endometrium and Myometrium Dysfunction in Adenomyosis
Source: Front Genet. 2020 Jul 3;11:716. doi: 10.3389/fgene.2020.00716 (PMC7350935; doi:10.3389/fgene.2020.00716)

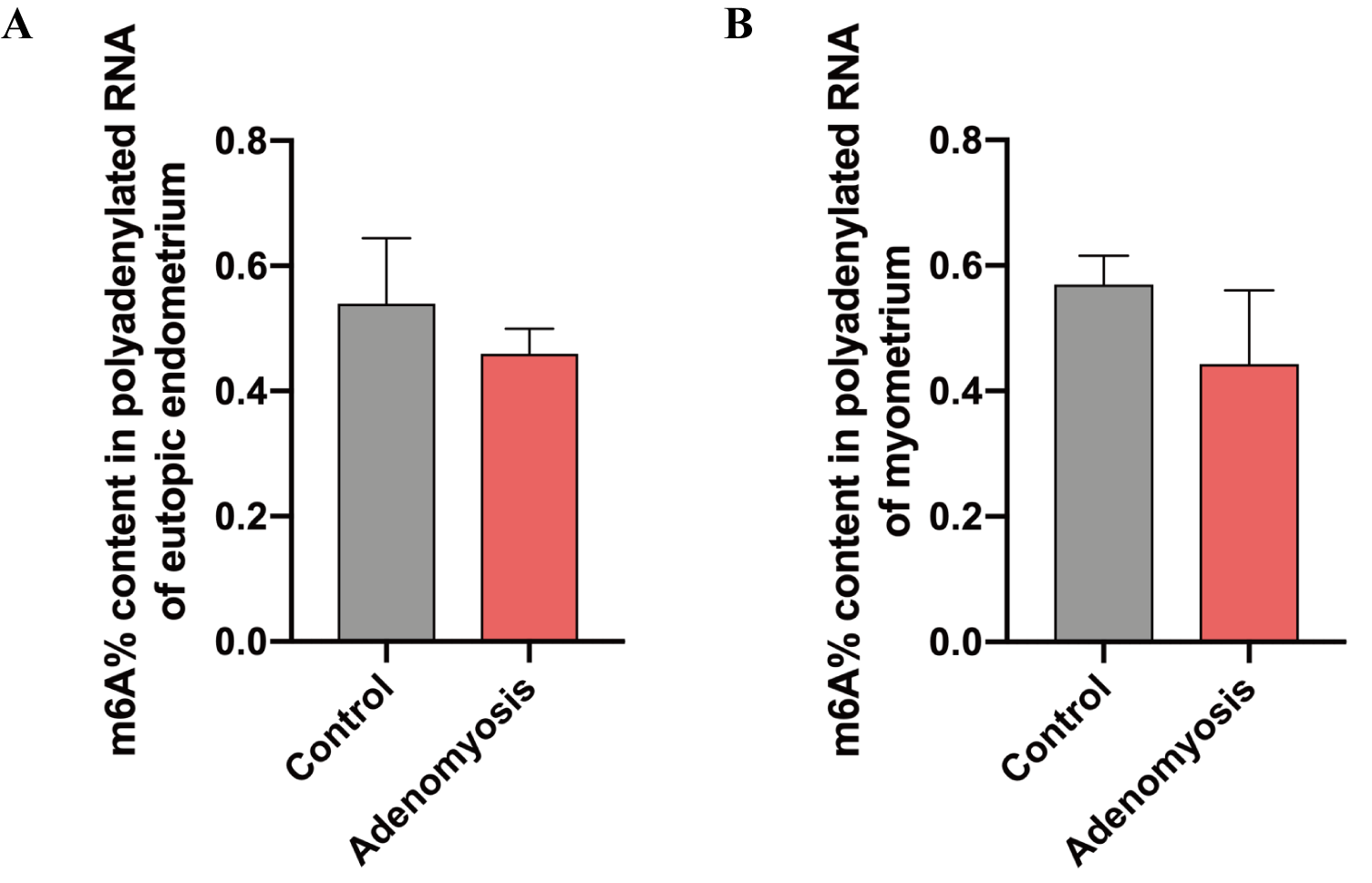

Supplement: FIGURE S1 — m6A% content in the polyadenylated RNA of eutopic endometrium and myometrium of women with and without adenomyosis. (A) Eutopic endometrium; (B) Myometrium. n = 3 samples were analyzed in each group. [file Image_1.TIF]

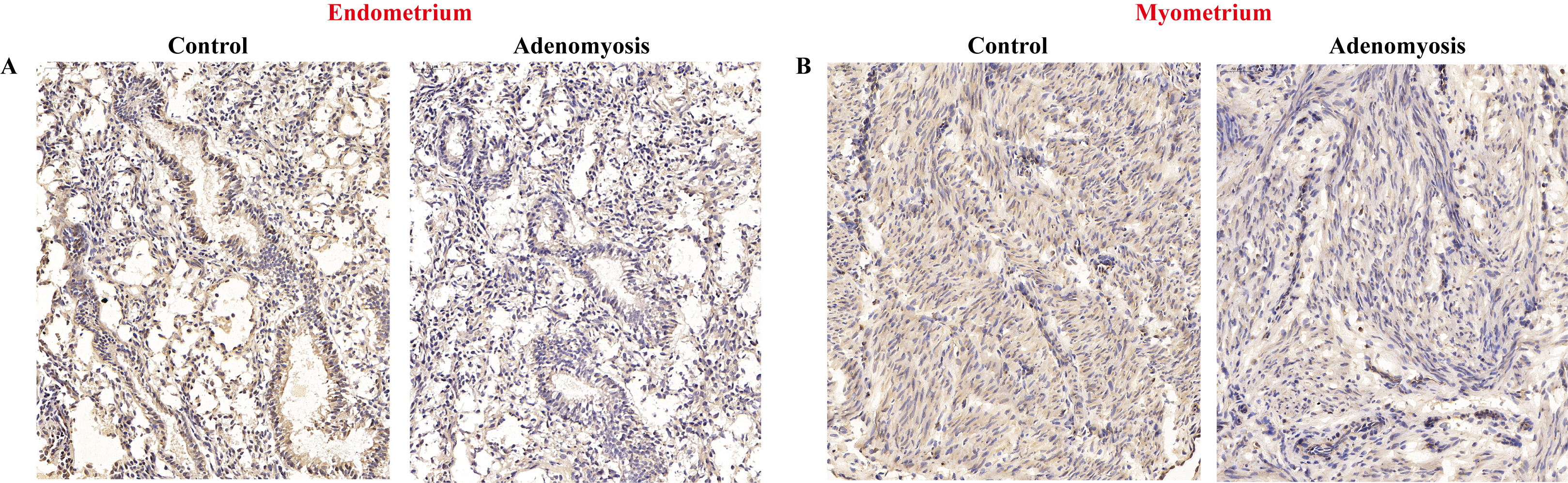

Supplement: FIGURE S2 — The IHC of METTL3 in the endometrium and myometrium of women with and without adenomyosis. (A) The endometrium of women with (right panel) and without adenomyosis (left panel). (B) the myometrium of women with (right panel) and without adenomyosis (left panel). Staining was assessed using Image J (n = 3 for each group) and scored on a scale of 0 (negative) to 3+ (high positive). Scale bars is 50 μm. The p-value was determined by a χ2-test. [file Image_2.TIF]

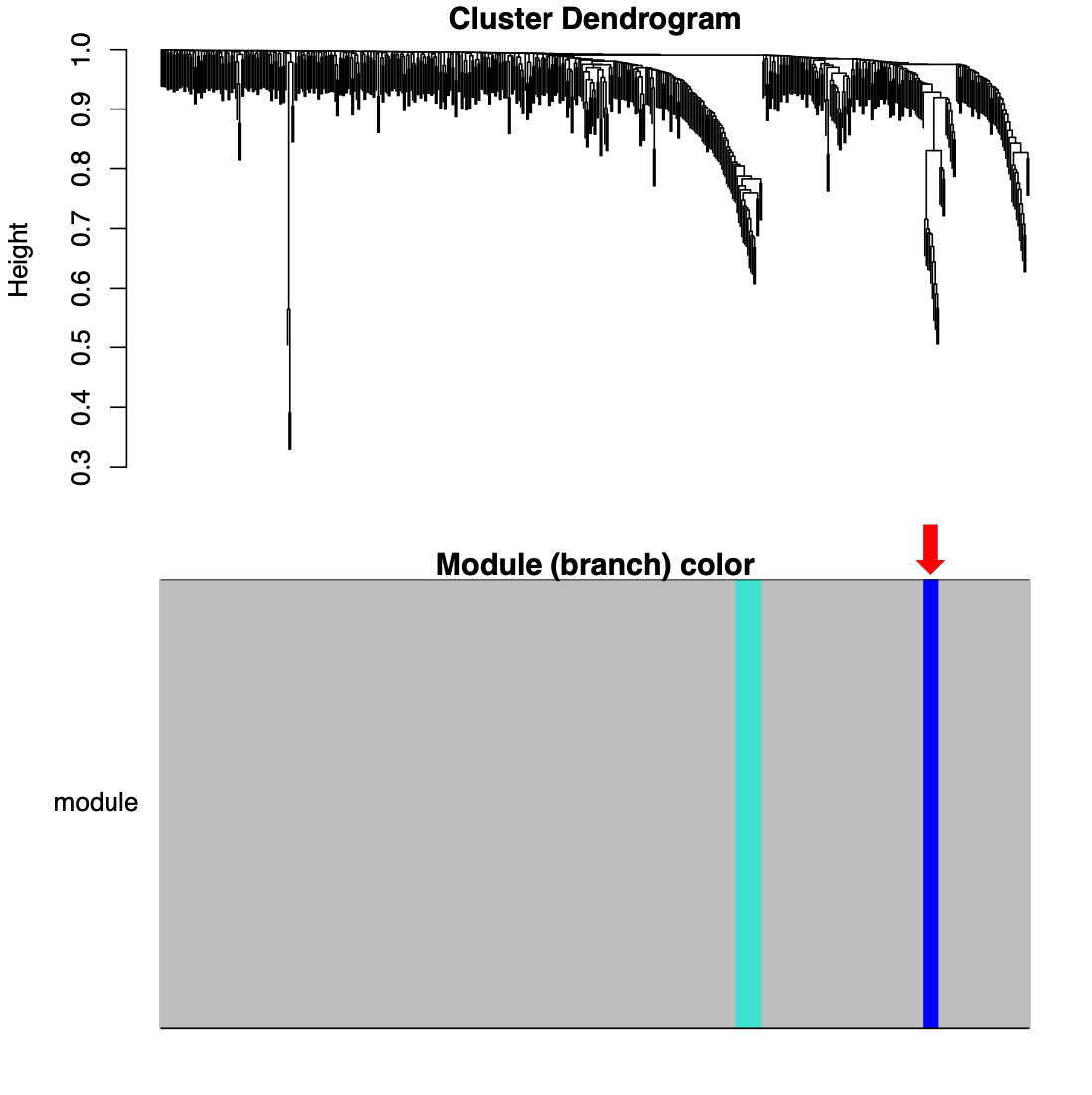

Supplement: FIGURE S3 — The WGCNA of DEGs in the myometrium of women with and without adenomyosis. Red arrow revealed the module that the differential expressed m6A RNA methylation regulators belongs to. [file Image_3.TIF]
