# Supplementary material for: m6A RNA Methylation Regulators Contribute to Eutopic Endometrium and Myometrium Dysfunction in Adenomyosis
Source: Front Genet. 2020 Jul 3;11:716. doi: 10.3389/fgene.2020.00716 (PMC7350935; doi:10.3389/fgene.2020.00716)
Supplement: Supplementary file 4 [file Table_1.docx]

**Table S1 The primer sequence of qRT-PCR**

| **Genes** | **Primer sequence** |
| --- | --- |
| METTL3 (human) Forward | 5’-TTGTCTCCAACCTTCCGTAGT-3’ |
| METTL3 (human) Reverse | 5’-CCAGATCAGAGAGGTGGTGTAG-3’ |
| METTL14 (human) Forward | 5’-GAACACAGAGCTTAAATCCCCA-3’ |
| METTL14 (human) Reverse | 5’-TGTCAGCTAAACCTACATCCCTG-3’ |
| ALKBH5 (human) Forward | 5’-CGGCGAAGGCTACACTTACG-3’ |
| ALKBH5 (human) Reverse | 5’-CCACCAGCTTTTGGATCACCA-3’ |
| FTO (human) Forward | 5’-ACTTGGCTCCCTTATCTGACC-3’ |
| FTO (human) Reverse | 5’-TGTGCAGTGTGAGAAAGGCTT-3’ |
| CDH3 (human) Forward | 5’-ATCATCGTGACCGACCAGAAT-3’ |
| CDH3 (human) Reverse | 5’-GACTCCCTCTAAGACACTCCC-3’ |
| SCN4B (human) Forward | 5’-TCTTCCTGCTCCCCGTAAC-3’ |
| SCN4B (human) Reverse | 5’-AATGCGTCACTGCTGTTGTAG-3’ |
| PLAC8(human) Forward | 5’-GTGTGACTGTTTCAGCGACTG-3’ |
| PLAC8 (human) Reverse | 5’-CTGCAACTTGACACCCAAGG-3’ |
| ZC3H13(human) Forward | 5’-GTGCCGTAACTGGCTGAAGA-3’ |
| ZC3H13 (human) Reverse | 5’-CCTTTACCACGAGGTGAAGGG-3’ |
| YTHDC1 (human) Forward | 5’-AACTGGTTTCTAAGCCACTGAGC-3’ |
| YTHDC1 (human) Reverse | 5’-GGAGGCACTACTTGATAGACGA-3’ |
| IGF1 (human) Forward | 5’-GCTCTTCAGTTCGTGTGTGGA-3’ |
| IGF1 (human) Reverse | 5’-GCCTCCTTAGATCACAGCTCC-3’ |
| DDT (human) Forward | 5’-GGAGCTGGACACGAATTTGC-3‘ |
| DDT (human) Reverse | 5’-ACTACGCCGATGGAGGAGAT-3’ |
| ACTIN (human) Forward | 5’-GGGAAATCGTGCGTGACATTAAG-3’ |
| ACTIN (human) Reverse | 5’-TGTGTTGGCGTACAGGTCTTTG -3’ |
| CNN3 (human) Forward | 5’-GAAGAAGGTCAACGAGTCCTCA-3’ |
| CNN3 (human) Reverse | 5’-AGTCTGAACCTGGGTCATGTT-3’ |
| EGR2 (human) Forward | 5’-TCAACATTGACATGACTGGAGAG-3’ |
| EGR2 (human) Reverse | 5’-AGTGAAGGTCTGGTTTCTAGGT-3’ |
